# Beef heifer fertility: importance of management practices and technological advancements

**DOI:** 10.1186/s40104-020-00503-9

**Published:** 2020-10-01

**Authors:** Sarah E. Moorey, Fernando H. Biase

**Affiliations:** 1 Department of Animal Science, University of Tennessee, Knoxville, TN USA; 2 Department of Animal and Poultry Sciences, Virginia Polytechnic Institute and State University, 175 West Campus Drive, Blacksburg, VA 24061 USA

**Keywords:** Beef cattle, Cow-calf, Genomics, Infertility

## Abstract

The development of replacement heifers is at the core of cow-calf beef production systems. In 2020, the USDA, National Agricultural Statistics Service reported 5.771 million beef heifers, 500 pounds and over, are under development for cow replacement. A compilation of data from several studies indicate that between 85% and 95% of these heifers will become pregnant in their first breeding season. Several thousands of heifers being raised for replacement may not deliver a calf on their first breeding season and result in economic losses to cow-calf producers. Many management procedures have been developed to maximize the reproductive potential of beef heifers. Such approaches include, but are not limited to the following: nutritional management for controlled weight gain, identification of reproductive maturity by physiological and morphological indicators, and the implementation of an estrous synchronization program. The implementation of management strategies has important positive impact(s) on the reproductive efficiency of heifers. There are limitations, however, because some heifers deemed ready to enter their first breeding season do not become pregnant. In parallel, genetic selection for fertility-related traits in beef heifers have not promoted major genetic gains on this particular area, most likely due to low heritability of female fertility traits in cattle. Technologies such as antral follicle counting, DNA genotyping and RNA profiling are being investigated as a means to aid in the identification of heifers of low fertility potential. To date, many polymorphisms have been associated with heifer fertility, but no DNA markers have been identified across herds. Antral follicle count is an indication of the ovarian reserve and is an indicator of the reproductive health of a heifer. We have been working on the identification of transcriptome profiles in heifers associated with pregnancy outcome. Our current investigations integrating protein-coding transcript abundance and artificial intelligence have identified the potential for bloodborne transcript abundance to be used as indicators of fertility potential in beef heifers. In summary, there is an ongoing pressure for reducing costs and increasing efficiency in cow-calf production systems, and new technologies can help reduce the long-standing limitations in beef heifer fertility.

## Importance of reproductive efficiency in beef cattle production

A great portion of the expenses in cow-calf production systems is dedicated to the maintenance of healthy cows in productive condition. At the same time, approximately one third of cows removed from the beef herd are eliminated because of reproductive failure (~ 33%, NAHMS 2007–2008). Thus, reproductive inefficiency is a limiting factor for the sustainability of beef cattle production systems that leads to financial losses to cattle producers [1].

In cattle, female reproductive failure is assumed when animals do not become pregnant within the breeding season or do not maintain pregnancy to calving [2]. Major female-related causes of reproductive failure include improper health, reproductive and nutritional management, reproductive disorders, and genetics [3–6]. To mitigate some negative factors that impact reproduction, practices associated with cow herd nutrition, healthcare, and reproductive management have been established.

The overall value of a beef female is calculated as the sum of all cash earned over her lifetime minus all expenses. While considerable economic inputs are required to develop replacement heifers, the calves produced throughout a cow’s productive lifespan may repay the costs of development and annual maintenance. The payback period is the period of time required for replacement heifers to pay for their development. This period may vary based upon expenses and cow productivity, but may generally be expected that a female must produce 6 calves to pay for her development and maintenance expenses [7]. If the cow fails to calve just 1 year of her productive lifespan, more than 8 calves are required [7], with no recovery of the lost revenue [8].

Another way to consider the profitability of retaining and developing replacement heifers is to calculate net present value (NPV) [7]. Net present value is calculated by accounting for all costs and revenues of the animal over her productive lifespan. Though NPV is heavily influenced by management scheme and calf performance, the reproductive efficiency of a cow greatly influences her NPV. An 11-year-old cow that first calved at age 2 and produced a calf each following year has a higher NPV than a cow that failed to calve 1 year during her productive lifespan [7].

## Importance of first breeding season success in replacement heifers

**Fig. 1:**
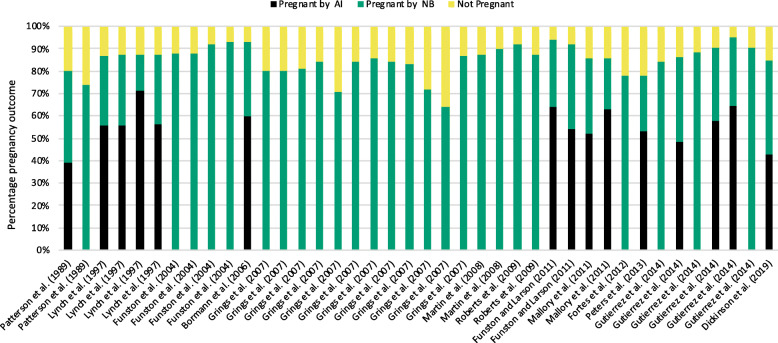
Pregnancy rates in beef heifers. *Y*-axis denotes percentage of pregnancy outcome, and *X*-axis indicates studies referenced. Multiple bars for the same reference indicate different experimental treatment within the same report

Heifer reproductive success in the first calving season is highly linked with lifetime reproductive efficiency [9–11]. A compilation of data from multiple studies demonstrated that first breeding season pregnancy rates in beef heifers range from 64 to 95% under natural breeding (NB) alone or the combination of artificial insemination (AI) followed by NB ([12–23] (Fig. 1)). Altogether, an average of 85% of heifers become pregnant by the completion of the breeding season. By comparison, first service conception rates to artificial insemination are lower and range from 36% to 69% [12, 14, 17, 18, 20, 21, 23, 24]. Our recent analysis of breeding records from 7 yrs (2011–2017) indicated that 43%, 42%, and 15% of heifers became pregnant by AI, NB, or failed to become pregnant during their first breeding season, respectively [23].

Under current production systems, the most efficient scenario occurs when replacement heifers conceive early in the breeding season. During their first breeding season, ~ 67%, ~ 26%, and ~ 7% of heifers that become pregnant are likely to calve during the initial 21 d, between d 22–42, and after d 42 of the subsequent calving season, respectively [11]. Heifers calving within the first 21 d of their first calving season remain in the productive herd longer and wean more total pounds of calf than their later calving counterparts [11]. In contrast, late breeding heifers contribute to a less efficient cow-calf production system due to reduced days postpartum to resume estrous cyclicity, reduced pregnancy rates in the subsequent calving season, and reductions in calf age and weaning weight [11, 25].

Considering the average pregnancy rate (85%) obtained from the data compiled in Fig. 1, and accounting for ~ 5.7 million heifers being developed as replacements in 2020 (data from National Agricultural Statistics Service, January 2020), one can estimate that approximately 3.3 million heifers will conceive in the first 21 d of the breeding season. Approximately 1.6 million heifers will conceive later in the breeding season, and over 800 thousand heifers will not produce a calf by ~ 23–27 months of age. These numbers underscore a critically large number of heifers that receive important farming resources but do not contribute to a long-term, sustainable production system.

Losses experienced from non-pregnant replacements are the result of opportunity costs of failing to market infertile heifers as feeder calves, wasted nutritional resources, and expenses of breeding and healthcare. If non-pregnant heifers were retained in the herd, such individuals would inevitably represent the negative impacts of a missed calving on NPV and payback period described above. Therefore, non-pregnant replacement heifers are often sold after a failed breeding season.

The costs of development, and reduced lifetime potential profitability, however, lead to a negative economic impact for cattle producers. Therefore, extra costs for heifer development due to losses when some heifers fail to become pregnant must be accounted for [26]. Depending on replacement heifer management system, these added costs can equate to ~$43 per replacement heifer developed to the time of pregnancy examination, as estimated by Hughes [26]. Considering the ~ 5.7 million heifers expected to enter replacement development in 2020, such cost might exceed $245 million nationwide. It must be pondered, however, that extra expenses caused by infertility can be reduced if the initial investment in heifer development is not extreme [13].

The economic impact of the reduced age of calves from late breeding heifers is also considerably high. Considering market prices of ~$1.65 per pound (USDA, Agriculture Marketing Service; Joplin Regional Stockyards; Feb 17, 2020; average prices of steer and heifer calves of ~ 450 lb.) and an average daily gain of ~ 1.90 lb. per day [27, 28], calves born at the midpoint of the second and third 21 d of the calving season would be worth approximately $131 less than calves born on the first day of the calving season. When this number is multiplied by the 1.6 million heifers expected to conceive late in their first breeding season, one can account for over $210 million lost by beef cattle producers due to late breeding heifers. These numbers underscore reproductive inefficiency among the major limiting biological functions significantly affecting the beef cattle industry.

The yearly cost of female infertility varies with the commodity value but remains unacceptably high under the current economic scenario. Since the early 1970s, it has been established that improving pregnancy rates is paramount for the development and maintenance of efficient and sustainable beef cattle production [29]. Since then, there have been major advancements to our understanding of the reproductive physiology of beef heifers and the identification of means to address reproductive inefficiency.

## Management practices to improve beef heifer reproductive success

The proper selection and development of replacement heifers enhances the likelihood that heifers entering development programs will conceive early in the breeding season followed by increased stayability [30]. Management strategies aimed at increasing first breeding season reproductive success are discussed below, and many are targeted towards increasing the percentage of heifers reaching puberty before the start of the breeding season. Such practices include the selection of older and heavier heifers at weaning [31], nutritional management of heifers to reach a defined percentage of their mature bodyweight by the start of the breeding season [16, 22], reproductive tract scoring to screen heifers for puberty ~ 30 d before the start of the breeding season [18], the implementation of a progestin-based estrous synchronization protocol [18, 32], and the incorporation of expected progeny differences (EPDs) to select heifers with increased genetic merit for fertility.

### Age of heifers

The selection of replacement heifers that are born early in the calving season is an essential step to optimizing overall reproductive success. It is expected that early born heifers will enter the breeding season with increased morphological and physiological maturity than their younger herd mates.

In a study by Funston and colleagues, heifers born in the first 21 d of the calving season were heavier at pre-breeding than heifers born in the second or third period of the calving season (296, 292 and 276 kg, respectively, [31]). Additionally, 70% of early born heifers were cycling by the start of their first breeding season, compared to 58% and 30% of heifers born in the second and third 21-d period, respectively. As a consequence, older heifers presented greater pregnancy rates (90%) compared to 86 and 78% for heifers that were born in consecutive 21-d windows of the calving period, respectively [31]. Our analysis of breeding records from Angus × Simmental crossbred heifers indicated that heifers older than 368 d of age at the beginning of the breeding season had 87.5% chance of becoming pregnant within 90 d compared to a 12.5% chance if the heifer was younger [23].

Heifers from different breeds reach puberty at different ages, ranging from 10 to 14 months, with crossbred heifers usually displaying estrus at an earlier age than purebreds [33–37]. These investigations also revealed that within a cohort of heifers of similar genetic make-up, some individuals will reach puberty early or late relative to their counterparts. Directly related to their age and physiological maturity, among cycling heifers, older heifers that are bred on their third estrous cycle present greater pregnancy rates (78%) relative to counterparts that are bred on their first estrous cycle (57%; [37]). Additionally, heifers entering the breeding season before reaching puberty or after one estrous cycle had reduced calving rates within the first 21 d of their first calving season compared to heifers experiencing at least 2 cycles before the onset of breeding [38].

Older heifers have a greater chance to become pregnant in their first breeding season. Nonetheless, it is critical that an appropriate balance is achieved for heifers to calve around 24 months of age, as these individuals will have a greater overall calving output relative to later breeding heifers [11, 39].

### Nutritional management of heifers

Appropriate nutritional status is essential for reproductive success in cattle. Energy restriction delays the ever critical onset of puberty in beef replacement heifers [40, 41]. Furthermore, inadequate energy consumption, as exhibited by low body condition score, reduces pregnancy success in beef cows throughout their productive lifespan [42]. By contrast, heifers experiencing higher levels of nutrition and adequate weight gain prior to the first breeding season experience increased reproductive success in their first and subsequent calving seasons [43, 44]. To this end, heifer development programs have been established for beef cattle producers to provide adequate nutrition for heifers to attain puberty and high reproductive success in their first breeding season. Cattle farms in different regions have varied sources of nutrients available for heifer development, and these feedstuffs have seasonal availability. Thus, the impact of the timing of weight gain on first breeding season pregnancy outcome has been evaluated.

No statistical differences in the percentage of heifers reaching puberty, becoming pregnant in the first or second 21 d of the breeding season, or conceiving by the end of the breeding season were observed among heifers managed to gain at a steady rate (0.45 kg/d), to gain none and then rapidly (0.91 kg/d), or to gain rapidly (0.91 kg/d) and then none during development from 45 d post-weaning to the start of the breeding season [45]. Heifers developed at a steady rate, however, had first service pregnancy rates of 62% as compared to 47% and 35% in fast-slow or slow-fast gaining heifers, respectively [45]. In a similar study, heifers that gained 0.11 kg/d initially, followed by 0.91 kg/d had similar first service conception rates and overall pregnancy rates when compared to heifers developed to gain weight at a constant 0.45 kg/d throughout the peri-pubertal period [20]. Nutritional management of heifers to gain weight in a stairstep fashion (fast gain, followed by slow gain, followed by fast gain immediately before breeding) yielded similar breeding season pregnancy rates as developmental programs with consistent gains [35, 46].

The timing of weight gain has minimal consequence for heifer fertility, but the weight a heifer reaches by the start of her first breeding season heavily impacts her reproductive success. Patterson and others demonstrated greater pregnancy rates when heifers reached 65% versus 55% of their mature body weight by the start of the breeding season [22]. Since then, reduced rates of puberty, but no difference in breeding season pregnancy rates have been reported in heifers managed to reach 55–56% versus 58–60% of their mature bodyweight [13, 16, 17]. Pregnancy rate to artificial insemination tended to be reduced in heifers developed to 55% [13], but was not reduced in heifers developed to 56% of mature body weight [17]. The development of heifers to 50% versus 55% of mature bodyweight also yielded no difference in overall 45-d breeding season pregnancy rates, but significantly delayed the date of first calving [19].

A large body of data reinforce the concept that heifers should be developed to reach a minimum percentage of their anticipated mature body weight by the start of the breeding season. It must be noted, however, that the target weight depends on heifer genetic makeup [47], nutritional management program, and breeding protocols utilized.

Inarguably, the feed source must be accounted when developing heifers to a target weight tailored to a particular cow-calf operation. For instance, the utilization of pasture, dormant range, or crop residues may provide valuable options for heifer development outside of the feedlot. Overall feed costs, total development costs, and net costs per pregnant heifer were significantly lower when heifers were developed to a lower bodyweight on forage diets than when heifers were developed solely in a dry lot [17, 48].

### Implementation of reproductive tract scores

The physiological and morphological maturity of the reproductive system is achieved as heifers attain puberty, but not all animals reach appropriate developmental status by the beginning of the breeding season. A reproductive tract scoring system ranging from 1 (pre-pubertal, infantile tract) to 5 (pubertal, corpus luteum present) was developed to categorize heifers according to uterine and ovarian development as determined by rectal palpation [49]. Usually, reproductive tract scoring is performed four to six weeks before the start of the heifer’s first breeding season and has become a tool to indicate the reproductive readiness of beef heifers.

**Fig. 2:**
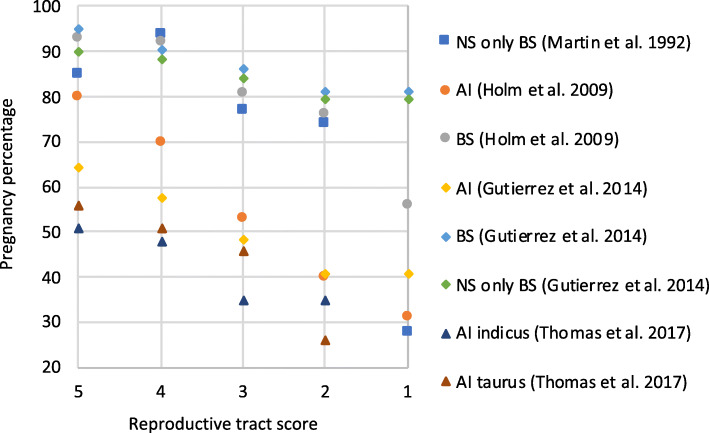
Pregnancy rates in beef heifers of different reproductive tract scores. *Y*-axis denotes pregnancy percentage, and *X*-axis denotes reproductive tract score categories. AI: artificial insemination, BS: breeding season, NB: natural breeding, indicus: *B. indicus*, taurus: *B. taurus*

Several independent reports have demonstrated that there is a strong, nearly linear relationship between reproductive tract score and pregnancy rates (Fig. 2). Lower scores (1 and 2) are consistently associated with lower pregnancy rates, whereas scores 4 and 5 indicate heifers that are cycling and therefore have greater pregnancy rates whether bred by AI alone or following a breeding season of AI followed by natural service [18, 23, 24, 47, 50]. Most cattle operations in the US may expect a majority of heifers to reach a reproductive tract score greater than 3 by the start of the breeding season. Reproductive tract scoring, however, remains an important tool to identify reproductively immature heifers or morphological abnormalities prior to breeding.

### Implementation of a progestin-based protocol for synchronization of estrus

Progestins can be used to induce puberty in peripubertal heifers and were initially used with estradiol to simulate the hormonal changes associated with the acquisition of puberty [51, 52]. Such changes begin with the increased progesterone levels associated with pubertal development in heifers [53]. The utilization of a progestin mimics this rise in progesterone and then allows for increased luteinizing hormone pulse frequency and desensitized negative feedback effects of estradiol on gonadotropin releasing hormone (GnRH) secretion [54, 55]. Therefore, peripubertal heifers experience increased follicular growth and estradiol production associated with fertile estrus and ovulation [54, 56].

There is an additional benefit from progestin-based protocols, whether through the utilization of a controlled internal drug release (CIDR; [57]) insert or melengestrol acetate (MGA; [58]). Such protocols synchronize ovulation in heifers and allow all heifers to be inseminated on day one of the breeding season. Overall, progestin based synchronization programs have a positive influence on heifer calving date and breeding season pregnancy rates [18, 19, 32, 59].

## The genetic basis of heifer fertility

Genetic selection is used to improve beef cattle populations for many production related traits. Relatively fast genetic progress can be achieved with traits such as growth rate and carcass quality because of their moderate to high heritability [60–62]. By contrast, the heritability of traits directly related to female reproduction is lower, and thus the rate of genetic change in fertility traits based upon genetic selection is much slower relative to growth and carcass traits. Despite low heritability, models are being developed utilizing genetic parameters to select beef cattle for the improvement of heifer fertility.

Pregnancy rate is a common trait utilized when evaluating fertility. Interestingly, the genetic correlation between yearling pregnancy rate and lifetime pregnancy rate is high, namely 0.92–0.97 [9, 10]. These findings support a genetic link between reproductive success in the first breeding season and productive lifespan, however the genes and genetic models of this correlation are yet to be unveiled.

The genetics of heifer pregnancy rate, or the likelihood of pregnancy within the first breeding season, is valuable to select heifers with increased genetic merit for pregnancy success. Genetic progress is limited because the heritability of heifer pregnancy rate ranges from 0.07 to 0.20 [12, 14, 61, 63–66]. First-service conception rate is another trait evaluated when considering heifer genetic merit for fertility. First-service conception rate identifies animals conceiving to their first service separately from animals conceiving later in the breeding season. The heritability of first-service conception in heifers is also low, ranging from 0.03 to 0.18 [12, 14, 61]. Altogether, diverse reports consistently indicate that pregnancy in beef heifers is controlled by a small portion of the additive component of a heifer’s genetic makeup.

Beef cattle production systems have greatly benefited from heterosis, but the investigations of heterosis on heifer fertility are scarce. Cundiff and others identified that crossbred heifers had 6.6% greater conception rate to natural service followed by 6.4% increase in calf crop weaned [67]. MacNeil and others observed that purebred or linecross heifers presented 76.2% and 79.4% pregnancy rates, respectively, but both groups had similar calf birth rates at 77% [68]. The effect of heterosis on heifer pregnancy is uncertain, but crossbreeding does influence heifer prebreeding weight and anticipated puberty onset [47].

## Current and emerging technologies for assessing fertility in heifers

The proper development of replacement heifers and the utilization of expected progeny differences for traits such as heifer pregnancy, first service conception rate, stayability, and scrotal circumference to select animals with superior genetics for fertility can improve heifer pregnancy rates. The impact of these means of selection and development eventually reach a plateau. Therefore, more detailed analyses of the phenotypic, physiological, and genetic components of heifer fertility are necessary. To this end, studies examining differences in genotypes, transcriptome profiles, and physical indicators of the ovarian reserve have been explored. As such, scientists have begun to reveal deep variations in otherwise phenotypically normal heifers of similar genetic background with remarkable contrasts in fertility potential. There is exciting opportunity for the utilization of many of these approaches to not only increase understanding of heifer fertility, but to identify additional parameters for the selection of highly fertile heifers.

### Antral follicle count

There is evidence that the selection of highly fertile heifers as replacement females may be improved with selection based upon antral follicle counts [69, 70]. In cattle, the oocyte and its surrounding follicle develop during fetal growth, with the presence of primordial follicles occurring by day 74–110 of gestation [71, 72]. Follicles remain quiescent at the primordial follicle stage until they are activated to the primary follicle stage and progress into the pre-antral and antral stages of follicular development [71, 72]. Antral follicles are then recruited into follicular waves that occur throughout the bovine estrous cycle [73]. The number of antral follicles present during a follicular wave can be determined by ultrasonography, in which the number of follicles ≥3 mm is reported as the antral follicle count (AFC).

Antral follicle counts are highly variable among animals, yet highly repeatable within an individual animal, allowing animals to be classified according to AFC [74]. Furthermore, AFC accurately depicts the ovarian reserve of cattle, in which animals with a low AFC possess less healthy primordial, pre-antral, and antral follicles compared to animals with high AFCs [75].

The ovarian reserve is related to fertility in female mammals. Cows with high AFC had higher pregnancy rates and shorter postpartum periods than animals with low AFCs [76]. Furthermore, AFC is associated with luteal and uterine function in cattle, and increased AFC was associated with higher reproductive success in beef heifers [69, 70]. A study of 47 young adult beef cattle and late lactation dairy cattle revealed that animals with low AFC had poorer endometrial development, followed by progesterone concentrations 30–50% lower than animals with high AFC [77].

Differences in oocyte competence have also been observed between animals with high versus low AFC. Ireland et al. [75] reported greater abundance of cathepsin mRNA in cumulus cells and increased intrafollicular estradiol concentrations in animals with low AFC, both of which are associated with reduced oocyte competence. Antral follicle counts hold great promise for improving replacement heifer selection criteria as they are determined though non-invasive procedures and are correlated with reproductive success in cattle.

### DNA polymorphisms

The ability to analyze thousands of single nucleotide polymorphisms (SNPs) allows researchers to investigate complex traits related through genome wide association studies (GWAS). Multiple studies have identified SNPs significantly associated with traits known to influence reproductive success in beef heifers, such as age at puberty [78, 79].

Peters and others identified twelve chromosomal regions associated with first service conception and six regions associated with heifer pregnancy [14]. Many of the regions containing SNPs associated with first service conception and heifer pregnancy corresponded to previously identified regions related to age at first corpus luteum [78]. Additionally, two regions on BTA2 and BTA8 were identified to have a relationship with heifer pregnancy. Single nucleotide polymorphisms identified on chromosome two were in close proximity to previously identified quantitative trait loci associated with differences in growth, carcass, lactation, and feed efficiency [62]. Such results support the importance of systems targeted research that considers the interconnectivity of animal body condition, growth, and reproductive outcome.

McDaneld and others identified SNPs associated with varied levels of reproductive success in *B. taurus* purebred, *B. taurus* × *B. taurus* crossbred, and *B. taurus* × *B. indicus* crossbred animals [80]. Due to the utilization of multiple populations of animals, individuals were either ranked for fertility upon reproductive outcomes in the first five breeding seasons or indicated as pregnant or open based on pregnancy success in the first or multiple breeding seasons. A single SNP on BTA29 achieved genome wide significance or nominal significance in some test populations. Interestingly, this SNP was within 786 kb of a SNP previously indicated to be associated with age at first identified corpus luteum in tropically adapted heifers [78]. Five additional SNPs on BTA1, BTA5, and BTA25 were identified at a suggestive level of significance in at least one population of animals. Single nucleotide polymorphisms on BTA5 coincided with previously reported SNPs associated with age at first corpus luteum, length of postpartum anestrous period, and the incidence of corpus luteum before calf weaning [78].

Additionally, presence of Y chromosome material was identified in low fertility and open classified heifers in the populations described above [81]. Approximately 18–29% of the heifers determined to have low fertility or failing to become pregnant, respectively, tested positive for segments of the bovine Y-chromosome.

Quantitative trait loci and gene networks were also identified in beef heifers previously classified as having high or sub-fertility based on d 28 pregnancy outcomes to serial embryo transfer [82]. Fourteen *loci* were strongly associated with heifer fertility, while eight *loci* displayed moderate association. Of these *loci*, five had positional candidate genes with previously indicated functions in fertility and uterine receptivity to pregnancy. One remarkable example is the gene kinesin family member 4A (*KIF4A*), which was located within the most significant locus associated with heifer fertility. Previous studies indicated elevated levels of *KIF4A* in endometrium samples collected on day seven post estrus in Simmental heifers who established pregnancy to embryo transfer following the next observed estrus compared to those that failed to establish pregnancy [83].

Investigations have also been conducted to understand fertility in *B. indicus* cattle, with emphasis on Nellore heifers. Many of these studies focused on identifying markers associated with heifers becoming pregnant by 14–16 months of age. Using a targeted approach, Camargo and others identified possible polymorphisms in the gene JY-1, an oocyte specific protein, associated with the probability of pregnancy by 16 months of age [84]. Several polymorphisms of interest for heifer fertility in zebu cattle were unveiled via GWAS analysis. Dias and others identified three haplotypes significantly associated with heifer pregnancy, which contained the genes fatty acid binding protein 4 (*FABP4*) and protein phosphatase 3 catalytic subunit alpha (*PPP3CA*) [85]. Focusing on chromosomic regions, two studies identified chromosome regions that explained as much as 8.91% [86] and 12.73% [87] of the variance in sexual precocity to become pregnant by 16 months of age and heifer pregnancy, respectively. Of note, both studies identified windows on chromosomes 5, 14, and 18, with a potential overlap on chromosome 14 [86, 87]. Takada and others focused on haplotypes encompassing 125 candidate genes and identified nine haplotypes with significant association with early pregnancy. Those haplotypes were located in the genes pregnancy-associated plasma protein-A2 (*PAPP-A2*), estrogen-related receptor gamma (*ESRRG*), pregnancy-associated plasma protein-A (*PAPP-A*), kell blood group complex subunit-related family (*XKR4*), and mannose-binding lectin (*MBL-1*) [88].

The Animal QTL database holds curated and compiled data on hundreds of DNA markers associated with diverse traits in livestock, including cattle [89]. The database currently has information on 56 markers associated with heifer pregnancy rate (Table 1). Throughout this selected data from the Animal QTL Database, and data from studies not identified in the database, it is important to notice that there is no clear redundancy of markers identified across studies. This observation points to the critical aspect of the replicability of the findings across populations [91] in addition to the complexity and most likely omnigenic nature of fertility.

**Table 1:** Quantitative trait *loci* present in the Animal QTL Database associated with beef heifer pregnancy^a^

QTL ID	Chr	Range, cM	Flank mark A	Peak mark	Flank mark B	Reference	Candidate gene symbol
137,399	1	Na	Na	rs108940570	Na	Regatieri et al. [90]	*APP*
151,129	1	119.14–120.06	rs136647907	Na	rs133111309	Júnior et al. [87]	Na
22,901	2	38.77–39.71	rs42919869	Na	rs43307553	Peters et al. [14]	Na
151,122	2	49.04–49.84	rs42509691	Na	rs134051905	Júnior et al. [87]	Na
151,125	2	52.56–53.43	rs133912634	Na	rs134084039	Júnior et al. [87]	Na
151,131	3	2.94–3.91	rs109945234	Na	rs42368646	Júnior et al. [87]	Na
22,902	4	3.97–4.89	rs110197100	Na	rs110954467	Peters et al. [14]	Na
151,119	5	Na	rs42917128	Na	rs136339681	Júnior et al. [87]	Na
107,840	5	10.23–11.66	Na	Na	Na	Irano et al. [86]	Na
108,449	5	18.49–19.71	Na	Na	Na	Irano et al. [86]	Na
151,128	5	56.06–57.01	rs110797637	Na	rs137576699	Júnior et al. [87]	Na
151,121	5	78.99–80.01	rs137127461	Na	rs109435449	Júnior et al. [87]	Na
151,113	5	80.05–81.09	rs109437025	Na	rs110687761	Júnior et al. [87]	Na
151,114	5	84.41–85.50	rs42561706	Na	rs137385583	Júnior et al. [87]	Na
151,115	5	88.95–89.84	rs110496647	Na	rs136544553	Júnior et al. [87]	Na
151,124	5	89.94–90.94	rs110450288	Na	rs133794376	Júnior et al. [87]	Na
119,777	6	12.13–13.29	Na	Na	Na	Irano et al. [86]	Na
57,465	6	28.43–28.44	rs134077806	Na	rs134383126	Dias et al. [85]	*PPP3CA*
57,466	6	28.54–28.54	rs109697066	Na	rs137526343	Dias et al. [85]	*PPP3CA*
119,778	7	3.77–4.65	Na	Na	Na	Irano et al. [86]	Na
119,779	7	49.92–50.82	Na	Na	Na	Irano et al. [86]	Na
22,903	8	0.40–1.10	rs110007458	Na	rs111021990	Peters et al. [14]	Na
152,647	8	115.08–115.08	rs135042546	Na	rs110990932	Takada et al. [88]	*PAPPA*
22,904	10	103.21–104.31	rs43647342	Na	rs41657367	Peters et al. [14]	Na
22,905	13	99.13–100.27	rs110209373	Na	rs41660868	Peters et al. [14]	Na
151,116	14	28.67–29.98	rs41724652	Na	rs133297141	Júnior et al. [87]	Na
152,648	14	30.64–30.66	rs42646650	Na	rs134214692	Takada et al. [88]	*XKR4*
119,780	14	29.53–30.56	Na	Na	Na	Irano et al. [86]	Na
151,127	14	31.34–32.62	rs135852767	Na	rs42298467	Júnior et al. [87]	Na
151,118	14	36.75–37.97	rs41624840	Na	rs136805030	Júnior et al. [87]	Na
57,464	14	61.17–61.17	rs132819090	Na	rs109077068	Dias et al. [85]	*FABP4*
152,641	16	25.33–25.36	rs136930654	Na	rs132925189	Takada et al. [88]	*ESRRG*
152,642	16	25.65–25.67	rs133536959	Na	rs109979901	Takada et al. [88]	*ESRRG*
152,643	16	73.28–73.35	rs136672059	Na	rs109160879	Takada et al. [88]	*PAPPA2*
152,644	16	73.35–73.38	rs135370722	Na	rs132969356	Takada et al. [88]	*PAPPA2*
152,645	16	73.39–73.41	rs132814943	Na	rs42300953	Takada et al. [88]	*PAPPA2*
152,646	16	73.56–73.66	rs132776805	Na	rs41814719	Takada et al. [88]	*PAPPA2*
151,117	18	Na	rs136460244	Na	rs41891085	Júnior et al. [87]	Na
119,781	18	5.63–6.48	Na	Na	Na	Irano et al. [86]	Na
22,906	20	81.03–82.15	rs41959108	Na	rs110359079	Peters et al. [14]	Na
137,400	21	Na	Na	rs134589009	Na	Regatieri et al. [90]	Na
137,401	21	Na	Na	rs134601255	Na	Regatieri et al. [90]	*SETD3*
119,782	21	0.01–3.77	Na	Na	Na	Irano et al. [86]	Na
119,783	21	77.11–77.86	Na	Na	Na	Irano et al. [86]	Na
137,402	22	Na	Na	rs133503069	Na	Regatieri et al. [90]	*ARHGEF3*
151,123	24	61.41–62.40	rs109329309	Na	rs135881583	Júnior et al. [87]	Na
151,120	24	70.77–71.92	rs136828522	Na	rs137238317	Júnior et al. [87]	Na
119,784	27	1.83–2.91	Na	Na	Na	Irano et al. [86]	Na
152,649	28	50.38–50.38	rs136285814	Na	rs133640737	Takada et al. [88]	Na
31,164	29	18.28–18.48	Na	Na	Na	de Camargo et al. [84]	*JY-1*
31,165	29	18.28–18.48	Na	Na	Na	de Camargo et al. [84]	*JY-1*
31,166	29	18.28–18.48	Na	Na	Na	de Camargo et al. [84]	*JY-1*
31,167	29	18.28–18.48	Na	Na	Na	de Camargo et al. [84]	*JY-1*
151,130	29	33.02–34.37	rs134769207	Na	rs42172278	Júnior et al. [87]	Na
151,126	X	Na	rs134685381	Na	rs137716652	Júnior et al. [87]	Na
151,132	X	64.37–65.63	rs134673004	Na	rs134676523	Júnior et al. [87]	Na

^a^ The completeness of the database is dependent on the submission of data by researchers; Na: not available

## Promise for the development of bloodborne indicators of heifer fertility

Recent studies have demonstrated that the profiling of circulating biological features (hormones, metabolites, transcripts, or epigenetic marks on the DNA of circulating cells) is revealing of the physiological state of an individual [92]. The analysis of multiple layers of an individual’s molecular blueprint is likely key for the understanding of several complex traits, in a health context and otherwise [93]. The systemic profiling of circulating biological features is likely to also contribute to the understanding of infertility [94].

### Bloodborne mRNA profiles

Considering that the physiology of an individual is highly linked to molecular features circulating in the bloodstream and the relationships of peripheral blood natural killer cells with fertility in women, we reasoned that mRNA profiles of peripheral white blood cells (PWBC) may differ among beef heifers who became pregnant to AI, pregnant to NB, and failed to become pregnant in their first breeding season. Our first profiling of mRNA transcripts from heifers from different pregnancy outcomes revealed six DEGs (*ALAS2*, *CNKSR3*, LOC522763, *SAXO2*, *TAC3*, *TFF2,* FDR < 0.05) between heifers that became pregnant to AI and heifers that did not become pregnant [95]. In a follow up experiment, we identified 67 DEGs (FDR < 0.03) between AI-pregnant and non-pregnant heifers.

A natural question is whether we can use gene expression profiles to distinguish phenotypes. To this end, we analyzed our data using top scoring pair approach and revealed two gene pairs (C11orf54, *TAF1B*; *URB2*, ENSTAG00000039129) whose relative expression within heifers discriminated most AI-pregnant (10 out of 12) from the other heifers profiled [95]. In a subsequent study, we applied machine learning algorithms on data obtained from two breeding seasons. The data from year 2015/2016 was used to train the algorithms and the data from 2016/2017 was used for blind predictions and assessment of model accuracy. The heifers in the test data were classified with 100% of accuracy in 46.3% out of 2000 randomizations), followed by 53.1% correct classification for 10 out of 11 heifers [96]. Altogether, remarkable differences exist in the abundances of genes expressed in the PWBC of heifers of differing fertility outcomes, and there is strong indication that these differences are useful as predictive tools for pregnancy outcome.

### Circulating miRNA profiles

MicroRNAs (miRNAs) are a class of short, single stranded, non-coding RNAs that regulate gene expression post-transcription and impact several fundamental biological processes [97, 98]. While most of the functions are exerted within the cellular compartment, cells have the ability to export those molecules to the extracellular environment mostly packed within exosomes, microvesicles or associated with lipoprotein or protein complexes [99] and can be found in serum [100].

In an attempt to assess the predictive value of circulating miRNAs on first breeding season pregnancy outcome, we profiled small RNAs collected from plasma of 18 beef heifers on the day of fixed-time AI. One miRNA (miR-11995) was more abundant in plasma of AI-pregnant heifers than not-pregnant heifers, while no differences in miRNA expression were detected between NB-pregnant and not-pregnant or AI-pregnant and NB-pregnant heifers [96]. Such results indicate that expression profiles of miRNA alone may provide little indication of heifer fertility. A multilayered approach combining miRNA expression and relative coexpression with mRNA targets may be required to provide further insight into differences of replacement heifer fertility potential.

## Conclusion and future directions

The selection and management of highly fertile replacement heifers will greatly impact the future success of the worldwide beef cattle industry. As technologies allow cattle producers to more effectively identify animals that are sub- or infertile, those animals can be managed as feeder cattle and eliminated from the replacement heifer pool earlier in their productive lifespan. Less capital will be lost on the development costs of infertile individuals, and heifer pregnancy rates early in the first breeding season can be improved.

Scientists must identify parameters beyond phenotypic traits and traditional genetic predictions to improve the producer’s ability to retain only the most fertile individuals. While incorporation of AFC into replacement heifer evaluation may increase detection of lowly fertile animals, additional means to further determine heifer fertility potential must be identified. Single nucleotide polymorphism profiling of certain populations of animals has indicated potential genetic markers of fertility in heifers, however further understanding of differences in the transcription of mutated genes and their outcomes on heifer fertility beg for studies focused at the transcriptome and protein level. Recent studies have demonstrated remarkable differences in bloodborne mRNA of heifers with different reproductive outcome in their first breeding season. Most importantly, we have identified the potential for specific gene transcripts to be successfully utilized to classify heifers by pregnancy outcome.

Advancements of on the molecular phenotyping of fertile heifers at the systemic level may fill a gap in current understanding of the physiology of reduced fertility in beef heifers and form a basis from which additional studies aim to develop means to estimate fertility potential in beef heifers. It remains an important question, however, whether such biotechnology can be incorporated into cow-calf production systems and contribute to sustainable beef production.

## Data Availability

Data sharing is not applicable to this article as no datasets were generated or analyzed during the current study.

## Abbreviations

AFC: Antral follicle count
AI: Artificial insemination
ALAS2: 5′-Aminolevulinate synthase 2
BTA: *Bos taurus* autosome
CIDR: Controlled internal drug release
Chr: Chromosome
cM: Centimorgan
CNKSR3: CNKSR family member 3
DEGs: Differentially expressed genes
DNA: Deoxyribonucleic acid
EPD: Expected progeny difference
ESRRG: Estrogen-related receptor gamma
FABP4: Genes fatty acid binding protein 4
FDR: False discovery rate
GWAS: Genome wide association studies
kb: Kilobases
kg: Kilogram
KIF4A: Kinesin family member 4A
lb.: Pound
LOC: Locus
MBL-1: Mannose-binding lectin
MGA: Melengestrol acetate
miR: MicroRNA
miRNAs: MicroRNA
NAHMS: National Animal Health Monitoring System
NB: Natural breeding
NPV: Net present value
PAPP-A: Pregnancy-associated plasma protein-A
PAPP-A2: Pregnancy-associated plasma protein-A2
PPP3CA: Protein phosphatase 3 catalytic subunit alpha
PWBC: Peripheral white blood cells
QTL: Quantitative trait loci
RNA: Ribonucleic acid
SAXO2: Stabilizer of axonemal microtubules 2
SNPs: Single nucleotide polymorphisms
TAC3: Tachykinin Precursor 3
TAF1B: TATA-box binding protein associated factor, RNA polymerase I subunit B
TFF2: Trefoil factor 2
URB2: URB2 ribosome biogenesis homolog
USDA: United States Department of Agriculture
XKR4: Kell blood group complex subunit-related family
